# Role of Nicotinamide in the Pathogenesis of Actinic Keratosis: Implications for NAD^+^/SIRT1 Pathway

**DOI:** 10.3390/biom14121512

**Published:** 2024-11-27

**Authors:** Riccardo Belardi, Francesca Pacifici, Terenzio Cosio, Sara Lambiase, Ruslana Gaeta Shumak, Fabio Artosi, Antonia Rivieccio, Danilo Cavalloro, Elena Dellambra, Luca Bianchi, David Della-Morte, Elena Campione

**Affiliations:** 1 Clinical Laboratory Medicine Unit, Department of Experimental Medicine, University of Rome Tor Vergata, Via Montpellier 1, 00133 Rome, Italy; riccardo.belardi@students.uniroma2.eu (R.B.); francesca.pacifici@uniroma2.it (F.P.); 2 Department of Human Sciences and Quality of Life Promotion, San Raffaele Roma Open University, 00166 Rome, Italy; david.dellamorte@uniroma2.it; 3 Interdisciplinary Center for Advanced Studies on Lab-on-Chip and Organ-on-Chip Applications (IC-LOC), University of Rome Tor Vergata, 00133 Rome, Italy; 4 Dermatologic Unit, Department of Systems Medicine, University of Rome Tor Vergata, Via Montpellier 1, 00133 Rome, Italy; terenziocosio@gmail.com (T.C.); sara.lambiase@alice.it (S.L.); ruslanagaetashumak@gmail.com (R.G.S.); fabio.artosi994@gmail.com (F.A.); rivieccio.antonia94@gmail.com (A.R.); danilocavalloro@libero.it (D.C.); luca.bianchi@uniroma2.it (L.B.); 5 Department of Experimental Medicine, University of Rome Tor Vergata, Via Montpellier 1, 00133 Rome, Italy; 6 Laboratory of Tissue Engineering, Istituto Dermopatico dell’Immacolata, IRCCS, Via dei Monti di Creta, 104, 00167 Rome, Italy; e.dellambra@idi.it; 7 Department of Neurology, Evelyn F. McKnight Brain Institute, University of Miami Miller School of Medicine, Miami, FL 33136, USA; 8 Department of Biomedicine and Prevention, University of Rome Tor Vergata, Via Montpellier 1, 00133 Rome, Italy

**Keywords:** sirtuin 1 activity, actinic keratosis, NAD^+^

## Abstract

Actinic keratosis (AK) is a precursor to invasive squamous cell carcinoma, making early diagnosis and treatment essential to prevent progression. Among available therapeutic options, nicotinamide (NAM) has shown potential in reducing AK progression. NAM is a precursor of nicotinamide adenine dinucleotide (NAD^+^), which activates sirtuin (SIRT)1, a protein with anti-cancer properties. Although the role of SIRT1 in AK is still debated, no data currently exist on the systemic modulation of this protein in AK. Therefore, this study aims to evaluate whether NAM, by increasing serum NAD+ levels, may promote SIRT1 activation in peripheral blood mononuclear cells (PBMCs) in AK patients. Thirty patients were enrolled and treated with NAM for 24 months. Hematological, biochemical, and skin condition assessments were conducted, alongside the measurement of SIRT1 and NAD^+^ levels. A decrease in basophils, monocytes, total cholesterol, and blood glucose levels was observed in the study group, along with a reduction in AK lesions. Notably, NAM treatment significantly enhanced serum NAD^+^ levels, and nuclear SIRT1 activity in PBMCs. In conclusion, NAM administration significantly reduced AK progression in a NAD^+^/SIRT1-dependent manner, supporting its role as a chemopreventive agent in AK management.

## 1. Introduction

Actinic keratosis (AK) is characterized by intraepithelial proliferation of atypical keratinocytes, which may progress into invasive squamous cell carcinomas (iSCCs) [1], and is therefore regarded as a precancerous lesion [2]. The incidence of AK is approximately 10% among individuals with light skin under the age of 30 but exceeds 80% after the age of 60 [3]. The progression rate to iSCC has been estimated at 0–0.075% per year for individuals without a prior history of non-melanoma skin cancer (NMSC) history, and 0–5% for those with a history of NMSCs [4,5]. Key risk factors include Fitzpatrick skin types I and II, advanced age, childhood sunburns, immunosuppression, and excessive sun exposure [6]. Among these, ultraviolet radiation (UVR) is particularly critical in the pathogenesis of AKs and their surrounding photodamaged skin, often referred to as the cancerization field (FC). Cumulative UVR exposure leads to genetic alterations, initially causing subclinical atypia and potentially advancing to keratinocyte neoplasms [6].

AKs are typically found on sun-exposed areas, including the face, bald scalp, auricles, back of the hand, and décolleté. While young individuals generally present isolated lesions, older individuals tend to develop multiple lesions across broader areas of sun-damaged skin [6]. Treatments for FC are often based on risk/progression factors such as lesion count, degree of photodamage, immunosuppression, and history of NMSC [6]. Additionally, non-invasive imaging techniques, including dermoscopy and reflectance confocal microscopy (RCM), are valuable for diagnosis and for monitoring treatment efficacy [7]. Due to the potential progression of AK to iSCC, accurate diagnosis and timely treatment are critical [4,5,8]. Early diagnosis facilitates a conservative approach and increases the likelihood of cure.

Therapies for AK are divided into preventive and curative strategies. Curative treatments, including cryotherapy, topical 5-fluorouracil (5-FU), imiquimod, tirbanibulin [6], and COX inhibitors like diclofenac or piroxicam are widely supported [9]. Cryotherapy, a straightforward and common treatment, has a success rate of 57–98.8%, although it may cause discomfort and post-treatment discoloration. Topical 5-FU, alone or combined with 10% salicylic acid, demonstrates an efficacy rate of around 75% with local irritation as a common side effect [10,11]. Imiquimod cream yields excellent results but may induce discomfort due to skin irritation and flu-like symptoms [12]. Piroxicam 0.8% topical formulation, combined with sunscreen, also effectively reduces AK lesions and FC in transplant patients or those on photosensitizing antihypertensive drugs [13,14,15].

Preventive AK therapy primarily involves sunscreen [16]. Moreover, nicotinamide, acitretin, and topical 5-fluorouracil (5-FU) are used in the chemoprevention, although some uncertainty persists in regard to which agent is most effective for immunocompetent versus immunosuppressed patients [17]. Oral nicotinamide (NAM) has demonstrated efficacy as a chemopreventive agent, particularly in patients with a history of two or more NMSCs [18,19]. NAM, an amide form of vitamin B3 found in foods like meat, fish, eggs, legumes, mushrooms, nuts, and grains, can also be synthesized through tryptophan metabolism [20]. Its chemoprotective effect is well established, and NAM administration has been shown to significantly reduce lesion count and size in AK patients, particularly in transplanted recipients [21]. Despite promising results, a 12-month placebo-controlled trial by Allen and colleagues found that NAM did not reduce the incidence of keratinocyte cancers or AK in immunosuppressed solid-organ transplant recipients, likely due to the altered immune function [22]. However, the mechanism underlying NAM’s effects remains unclear. NAM is a precursor of nicotinamide adenine dinucleotide (NAD^+^), which is crucial for adenosine triphosphate (ATP) production and serves as a coenzyme for various metabolic processes [23,24,25]. Beyond its role as a coenzyme, NAD^+^ is essential for maintaining genomic stability [23,24,26]. Physiological NAD^+^ levels protect against genomic instability and sun sensitivity in high-turnover tissues like the skin, where low NAD^+^ levels increased susceptibility to UV damage and skin cancer [27]. NAD^+^ also regulates various intracellular mechanisms, including cellular senescence, by serving as a substrate for sirtuins (SIRT), class III histone deacetylases that regulate the cell cycle, apoptosis, and energy homeostasis [28]. Although sirtuins are known to influence cell proliferation, their role in cancer remains controversial [29]. Elevated SIRT1 tissue levels, in fact, have been found to increase in NMSC, suggesting a potential tumor-promoting function [30]. However, SIRT1 expression is often reduced in other human tumors (such as glioblastoma, bladder carcinoma, and prostate carcinoma, among others) indicating it may, also, function as a tumor suppressor [31].

To the best of our knowledge, increased SIRT1 expression has mainly been analyzed in tumor tissue rather than in the serum of neoplastic patients. In agreement, in a study of patients with lung cancer, serum SIRT1 levels were lower than those in controls, suggesting that SIRT1 could exert tissue-specific function [32]. Maintaining adequate intracellular NAD^+^ levels and proper SIRT1 nuclear activity may therefore be critical for preventing DNA damage, genomic instability, and chronic inflammation, which contribute to increased cancer risk [33,34,35].

Based on this evidence, the aim of this present study was to evaluate whether NAM administration in AK patients could increase serum NAD^+^ levels and promote SIRT1 activation in peripheral blood mononuclear cells (PBMCs).

## 2. Materials and Methods

### 2.1. Study Population

This present study is an uncontrolled single-center and single-arm study, in which 30 patients, affected by previous NMSC, Aks, and FC were enrolled between 2021 and 2023 at the Dermatology Unit of the University Hospital of Rome “Tor Vergata”. These patients were treated with 1000 mg of oral nicotinamide (NAM) per day for 24 months. The sample size was calculated using two independent means, which were acquired from previous research [32,36].

During the baseline visit (Time 0, T0), all patients signed an informed consent and were screened for NMSC and AK history, number and localization of AK lesions, and hematological and biochemical parameters. During the follow-up visit (Time 24, T24), the patients were re-tested for all the parameters evaluated at T0 to evaluate the presence of new AKs or NMSC. The inclusion criteria for participants were (i) adult patients ≥ 18 years old (ii) with clinical and dermoscopic diagnosis of AK lesions. The exclusion criteria included patients who were (i) pregnant, (ii) immunosuppressed, or (iii) already undergoing preventive treatment for AK. At the beginning, prior to the commencement of treatment, as well as before each subsequent follow-up visit, a single physician initially assessed the lesions; however, this assessment was validated through clinical and dermatological evaluation by the specialized staff at the Dermatology Clinic. Moreover, when lesions appeared doubtful, a biopsy was performed.

This study was conducted in accordance with the ICH/GCP guidelines and in compliance with the Declaration of Helsinki. The experimental procedure was approved by the Institutional Review Board’s (IRB) Independent Ethical Committee “Tor Vergata” University Hospital (R.S. 139.23).

### 2.2. Blood Samples Collection and PBMCs Extraction

Whole blood and serum samples were collected from all subjects. The blood samples were obtained at the first clinical visit (T0) before any treatment and collected by peripheral vascular sampling using VACUETTE^®^ TUBE 8 mL CAT Serum Separator (Greiner Bio-One, Rome, Italy). Specifically, serum was obtained by centrifuging whole blood samples at 3500 rpm (3704× *g*) for 15 min, according to the standard operating procedures of the Laboratory Medicine Unit of the University Hospital of Rome “Tor Vergata”, and stored at −80 °C. Whole blood samples collected before NAM administration (T0) were used and compared with those obtained at the end of the study (T24). These samples were used for the extraction of peripheral blood mononuclear cells (PBMCs) using the Ficoll method [37]. Briefly, samples were layered on warmed Ficoll-Hypaque (GE-Healthcare, Little Chalfont, UK) and centrifuged at 2100 rpm (1334× *g*) for 20 min at room temperature. Then, the ring containing PBMCs was collected, washed with warmed PBS, and then centrifuged again. The pellet was freshly used to test the activity of Sirtuin 1 (SIRT1).

### 2.3. NAD/NADH and SIRT1 Immunoenzymatic Detection

Sera levels of different factors were assessed using a specific commercially available kit, following the respective manufacturer’s protocol. Specifically, NAD/NADH levels were determined using the colorimetric NAD/NADH Assay Kit (AB65348, Abcam, Cambridge, UK). NAD^+^ levels were indirectly evaluated by subtracting NADH from total NAD (NADt, represented by both NADH and NAD^+^) levels. SIRT1 levels were measured using the ELISA Human Sirtuin 1 ELISA Kit (MBS2601311, MyBiosource, Southern California, San Diego, CA, USA). The intensity and distribution of immunostaining from the different assays were assessed using a microplate reader (Multiskan FC, Thermo Fisher Scientific, Waltham, MA, USA).

### 2.4. SIRT1 Activity Assay

To determine the nuclear activity of SIRT1, PBMCs, extracted as previously reported, were processed using the Nuclear extraction kit (AB113474, Abcam, Cambridge, UK), following the manufacturer’s protocol. The freshly obtained nuclear extracts were immediately used for the fluorometric SIRT1 Activity Assay Kit (AB156065, Abcam, Cambridge, UK) according to the manufacturer’s protocol. The intensity and distribution of fluorescence were assessed using a fluorometric plate reader (Ascient Fluoroscan, Thermo Fisher Scientific, Waltham, MA, USA).

### 2.5. Statistical Analysis

The statistical analysis was performed by using GraphPad Prism version 9.1.1 (La Jolla, CA, USA). An unpaired two-tailed Student’s test, followed by Welch’s correction, was used for statistical analysis and significance. All data were expressed as mean ± SEM. Values of *p* < 0.05 were considered statistically significant.

## 3. Results

### Study Population Demographics and Clinical Parameters

The general demographic characteristics of the study population have been reported in Table 1. The mean age of our patients was 66.40 ± 12.00 years, with 76.5% being male. The most prevalent comorbidity within the population was hypertension, with an incidence rate of 52.9%, uniformly treated with the same pharmacological therapy.

In Table 2, we reported the pharmacological therapies related to the study population.

The general evaluation of biochemical parameters, including hematologic, renal, and hepatic functions, did not display significant variations during all the treatment period (Table 3).

However, we observed that NAM treatment significantly reduced the percentage of monocytes (*p* < 0.05) and basophils (*p* < 0.01) while still remaining within a physiological reference range. Moreover, we also reported a significant decrease in total cholesterol and blood glucose levels (*p* < 0.05), likely due to an increase in physiological activity following NAM administration.

**Figure 1:**
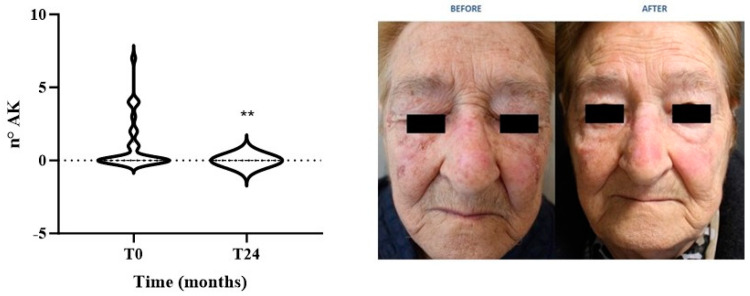
Evaluation of Actinic Keratosis (AK) lesion number in patients at T0 and T24, along with a representative patient’s image (** *p* < 0.005).

It has been demonstrated that NAM administration reduced the number and size of lesions in AK-transplanted patients [21]. In agreement, we evaluated the preventive effect of NAM as the mean AK number in each patient before and after treatment. As shown in Figure 1, we reported a significant decrease in the number of lesions at T24 in our study group (*p* < 0.005).

**Figure 2:**
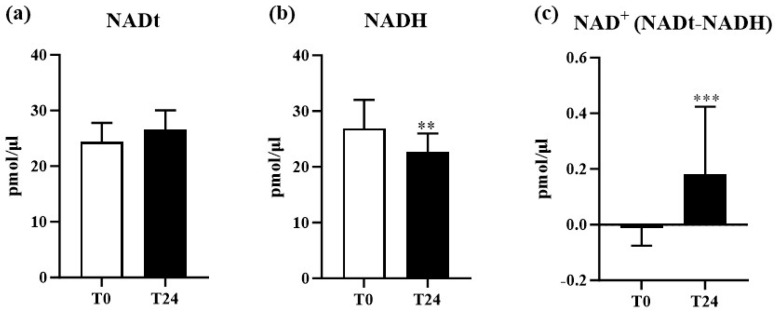
Sera levels of Nicotinamide Adenine Dinucleotide (NAD): (**a**) sera levels of Total NAD (NADt) in patients at T0 and T24; (**b**) sera levels of NAD + Hydrogen (NADH) in patients at T0 and T24; (**c**) sera levels of NAD Plus (NAD^+^) in patients at T0 and T24. ** *p* < 0.01; *** *p* < 0.001. pmol, picomoles.

Based on these findings, we move forward to analyze the molecular mechanism underlying NAM’s protective effect against AK. Since NAM is the precursor of NAD^+^ [23,24,38], we evaluated whether NAM administration influences NAD^+^ sera levels. Firstly, we measured NADt levels. As shown in Figure 2a, no significant modulation was observed in NADt sera levels throughout the study. However, although NADt levels did not change during NAM administration, we observed a significant reduction in NADH sera levels (*p* < 0.01) at T24 (Figure 2b), compared to T0. As expected, since NADt did not change, NAD^+^ circulant levels were significantly increased at the end of treatment compared to baseline (*p* < 0.001) (Figure 2c). These data suggest that NAM administration leads to an increase in NAD^+^ sera levels.

**Figure 3:**
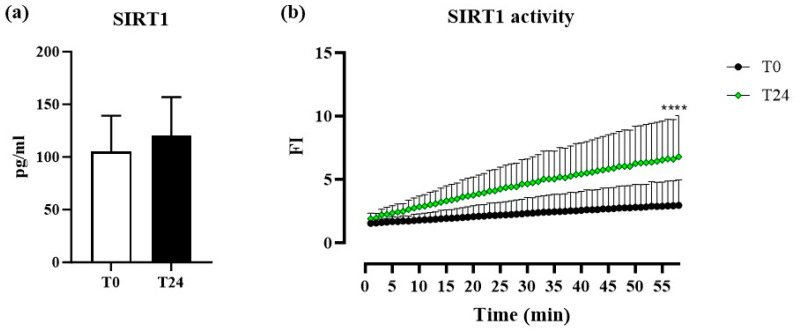
(**a**) Sera levels of NAD^+^-dependent deacetylases of the Sirtuin 1 (SIRT1) in patients at T0 and T24. (**b**) Nuclear activity of SIRT1 in peripheral blood mononuclear cells (PBMCs:) comparison of nuclear activity, monitored over 1 h, of Sirtuin1 (SIRT1), extracted from PBMCs, in patients at T0 and T24. (**** *p* < 0.0001). FI: fluorescence intensity.

It is well known that NAD^+^ plays a crucial role as a cofactor for the enzymatic activity of sirtuins, and specifically of SIRT1 [3,39]. These enzymes, in turn, regulate inflammatory and oxidative processes that are fundamental for maintaining cellular homeostasis and for the organism’s adaptive response to external and internal stimuli, particularly in tissues characterized by high cellular turnover, such as the skin [18]. Based on this evidence, we measured steady-state sera levels of SIRT1. As shown in Figure 3a, no significant variation at T24 was reported compared to baseline, suggesting that NAM administration did not influence the steady-state protein levels. Therefore, we investigated whether NAM administration, by increasing NAD^+^ levels, might modulate SIRT1 activity rather than its serum steady-state expression. In particular, since SIRT1 is enzymatically active when displaying a nuclear localization, we isolated nuclei from PBMCs extracted from the enrolled patients, and measured SIRT1 activity. As shown in Figure 3b, and as expected, nuclear SIRT1 activity at T24, showed a statistically significant increase (*p* < 0.0001) compared to T0. Based on this evidence, we can speculate that the administration of NAM increases NAD^+^ sera levels, which, in turn, impacts SIRT1 by increasing its nuclear activity.

## 4. Discussion

The results of this study provide significant insights into the effects of NAM administration in patients with AK. Our findings confirm NAM’s protective effects on AK lesions and indicate that NAM treatment leads to a notable increase in serum NAD^+^ levels without significantly altering total NAD levels. This is consistent with the understanding that NAM, as a precursor to NAD^+^, enhances NAD^+^ availability in the serum, potentially impacting several downstream cellular processes.

The evaluation of biochemical parameters revealed a reduction in the levels of both monocytes and basophils. Basophils are known to migrate into skin lesions in disorders such as atopic dermatitis, recruiting and polarizing monocytes, which drive pro-inflammatory response [40]. Although we did not assess basophil levels in the lesions, NAM administration may have inhibited basophil and monocyte recruitment to skin lesions by reducing their serum levels within physiological ranges.

NAM effects on AK lesions have been previously reported. In a randomized clinical trial by Drago and colleagues, 88% of AK transplanted patients experienced a significant reduction in both size and number of lesions compared to relative controls [21]. Similarly, we reported a significant reduction in the number of AK lesions in our patients, likely due to decreased inflammation driven by reduced basophils and monocyte levels.

The observed reduction in NADH levels alongside the increase in NAD^+^ levels suggests a shift in the redox state, potentially crucial in regulating cellular metabolism and maintaining homeostasis in high-turnover tissues such as the skin. NAD^+^ supports the enzymatic activity of several targets like the SIRT family, well-known modulators of inflammatory and oxidative processes, which underscores NAM’s therapeutic potential in AK management [3,39,41]. Chen et al. [16] demonstrated NAM’s role in enhancing DNA repair in UV-irradiated keratinocytes, thereby reducing AK formation and subsequent SCCs. Additionally, NAM’s increase in NAD^+^ levels, essential for the activity of enzymes like sirtuins, maintains genomic stability and reduces inflammation [16]. DNA repair processes require energy generated by glycolysis, β-oxidation, and other NAD^+^-regulated intracellular processes [42]. In our study, we observed an increase in NAD^+^ levels, and a decrease in both glucose and total cholesterol, suggesting an enhancement in energy transduction efficiency that may contribute to the beneficial effect of NAM on skin lesions.

Interestingly, while serum levels of SIRT1 did not change significantly following NAM administration, nuclear SIRT1 activity in PBMCs increased markedly. This finding suggests that NAM’s influence on SIRT1 is mainly nuclear, highlighting a potential mechanism by which NAM protects against DNA damage and genomic instability [33,34,35]. Some studies have shown varying SIRT1 responses across tissue types and cancer models, indicating the need for further research on SIRT1 modulation by NAM in AK patients [32,43,44,45]. In particular, it has been demonstrated that SIRT1 levels are increased in AK or NMSC lesions without reporting its localization. The aberrant localization of molecules, such as those involved in cell cycle progression or cell survival, may have a role in cancer development [46]. SIRT1 is mainly nuclear in non-tumorigenic cells but often cytoplasmic in tumorigenic cells (e.g., breast, prostate, lung) [45]. Therefore, it is possible to speculate that cytoplasmic (aberrant) SIRT1 localization promotes tumor growth, while nuclear localization may act as a tumor suppressor, as suggested by our findings.

Our evidence supports NAM’s potential chemoprotective effects via nuclear SIRT1 activity modulation, suggesting NAM not only serves as a preventive agent but also modulates key molecular pathways involved in cellular protection and repair [32,43,44,45,47]. To the best of our knowledge, this is the first study reporting NAM’s effect on steady-state serum SIRT1 activity in AK patients.

Some limitations of this study should be acknowledged. First, we did not measure Nicotinamide N-methyltransferase (NNMT) levels, which play a key role in NAD^+^ levels and influence its salvage pathway. NNMT methylates NAM, preventing its entry into the NAD^+^ salvage pathway, thus reducing NAD^+^ levels [48]. NNMT levels are also increased in skin cancer lesions, which may reduce NAM’s anti-cancer efficacy [49]. While we did not assess NNMT activity, we hypothesize that chronic NAM administration could be partially methylated by NNMT while also contributing to the NAD^+^ salvage pathway. It is also plausible that chronic NAM treatment may, at least in part, downregulate NNMT activation by the end of the study. Further study is needed to clarify NNMT’s role in NAM-treated AK patients.

Another limitation of our study is the small sample size and the lack of a control population without NAM supplementation. Larger clinical trials are necessary to confirm this preliminary evidence. Additionally, confounding factors, such as concomitant medications, could impact NAM’s effects. Increasing the sample size may enable subgroup analysis to better understand potential interaction with other therapies.

## 5. Conclusions

In conclusion, NAM administration appears to enhance NAD^+^ levels and nuclear SIRT1 activity, contributing to its protective effects in patients with AK. These findings warrant further investigation into the molecular mechanisms underlying the therapeutic benefits of NAM and the development of targeted strategies to optimize its use in clinical practice. Early recognition and accurate diagnosis of AK, combined with NAM administration, offer a promising approach for personalized patient management and improved clinical outcomes.

## Figures and Tables

**Table 1:** General demographic characteristics of the study population.

Variables	Total (*n* = 30)
Age (years)	66.4 ± 12.0
Gender:	
Male %	76.5
Female %	23.5
Comorbidities	
Cardiovascular Diseases	
Hypertension %	52.9
Atrial Fibrillation %	17.6
Dyslipidemia	
Hypercholesterolemia %	26.5
Hypertriglyceridemia %	8.8
Others	
Benign Prostatic Hypertrophy %	17.6
Diabetes %	11.8
Vitamin B Deficiency %	8.8
Hyperuricemia %	5.9
Gastroesophageal Reflux %	2.9
Hypothyroidism %	2.9
Psychiatric Disorders %	2.9

**Table 2:** Pharmacological therapy of the study population.

Pharmacological Therapy	%
Beta blockers	52.9
Statins	38.2
Alpha blockers	17.6
Anticoagulants	17.6
Diuretics	17.6
Insulin	11.8
Metformin	11.8
Proton Pump Inhibitors	8.8
Allopurinol	5.9
Hormonal analogues	2.9
Benzodiazepines	2.9
Vitamin B9	2.9

**Table 3:** General evaluation of biochemical and hematologic parameters.

Parameters	Time 0 (*n* = 30)	Time 24 (*n* = 30)	*p* Value *
WBC (cells × 10^3^/L)	6.7 ± 1.7	7.3 ± 3.3	0.4116
RBC (cells/mcL)	4.7 ± 0.5	4.6 ± 0.5	0.2886
Hb (g/dL)	14.6 ± 1.2	14.6 ± 1.5	0.8140
Hct (%)	43.1 ± 3.4	42.6 ± 3.7	0.6335
Plt (cells/mcL)	224.6 ± 56.8	233.3 ± 54.3	0.5577
Neut %	61.5 ± 7.5	63.5 ± 8.5	0.3552
Lymph %	28.1± 6.6	27.5 ± 8.2	0.7830
Mono %	7.3 ± 1.7	6.3 ± 1.3	0.0113 *
Eos %	2.3 ± 1.9	2.0 ± 1.4	0.5458
Baso %	0.8 ± 0.4	0.5 ± 0.3	0.0013 **
BUN (mg/dL)	41.5 ± 10.8	40.9 ± 11.0	0.8385
Glu (mg/dL)	100.1 ± 11.9	89.2 ± 18.7	0.0220 *
TB (mg/dL)	0.8 ± 0.4	0.8 ± 0.3	0.5801
TG (mg/dL)	123.7 ± 58.2	100.1 ± 43.6	0.0837
GGT (UI/L)	27.2 ± 6.8	27.6 ± 16.4	0.9067
AST/SGOT (U/L)	29.4 ± 18.3	27.4 ± 13.8	0.6316
ALT/SGPT (U/L)	30.0 ± 20.2	30.8 ± 21.1	0.8958
Cr (mg/dL)	1.0 ± 0.2	1.0 ± 0.4	0.8160
eGFR (mL/min)	74.6 ± 17.3	80.6 ± 23.0	0.2801
TC (mg/dL)	189.7 ± 36.7	174.0 ± 19.9	0.0393 *

WBC (White Blood Cells); RBC (Red Blood Cells); Hb (Hemoglobin); Hct (Hematocrit); Plt (Platelets); Neut (Neutrophils); Lymph (Lymphocytes); Mono (Monocytes); Eos (Eosinophils); Baso (Basophils); BUN (Blood Urea Nitrogen); Glu (Glucose); TB (Total Bilirubin); TG (Triglycerides); GGT (Gamma-Glutamyl Transferase); AST (Aspartate Aminotransferase); SGOT (Serum Glutamic-Oxaloacetic Transaminase); ALT (Alanine Aminotransferase); SGPT (Serum Glutamic-Pyruvic Transaminase); Cr (Creatinine); eGFR (estimated Glomerular Filtration Rate); TC (Total Cholesterol); * *p* < 0.05; ** *p* < 0.01.

## Data Availability

All data are reported in the manuscript and are available from the corresponding author.
